# Crossing the equator: a northern occurrence of the pygmy right whale

**DOI:** 10.1186/s40851-018-0117-8

**Published:** 2018-12-17

**Authors:** Cheng-Hsiu Tsai, James G. Mead

**Affiliations:** 1 Department of Life Science, National Taiwan University, Taipei, 10617 Taiwan; 2 Department of Vertebrate Zoology, National Museum of Natural History, Smithsonian Institution, Washington, D.C., 20013-7012 USA

**Keywords:** Cetacea, Mysticeti, Biogeography, Stranding

## Abstract

Here we document the first stranding record of the pygmy right whale in the Northern Hemisphere—on the coast of The Gambia, Africa (NE Atlantic Ocean, around latitude 13° N)—a location in stark contrast to its current distribution exclusively south of the equator. The original specimen is now missing and untraceable, but a photo found in the files of the Marine Mammal Program, Smithsonian Institution shows sufficient diagnostic features that allow it to be taxonomically identified as the pygmy right whale, *Caperea marginata*, including: small body size; streamlined overall body shape; generally dark skin coloration; arched rostrum along the lateral margin; triangular and narrow rostrum in dorsal view; lack of head callosities; some fringes on the dorsal surface of the tongue; small and relatively posteriorly positioned dorsal fin; and small and dark-colored flipper. On the whole, a stranding of the pygmy right whale in the Northern Hemisphere, although likely to be a chance event, calls for more detailed studies of how climate change and ocean currents affect the evolution and distribution (re-patterning) of marine mammals and, ultimately, the entire marine ecosystem.

## Introduction

The pygmy right whale *Caperea marginata* (Cetacea: Mysticeti) is one of the least known large and extant vertebrate species, due to its elusive nature and comparatively restricted distribution in the Southern Hemisphere [1, 2]. Similarly, *Caperea*-like fossils are extremely scarce and also mainly found in the Southern Hemisphere [3–5], except for two recent reports of fossil *Caperea* from the Pleistocene of Japan and Italy, respectively [6], indicating a more complicating scenario for the origin and evolution of marine mammal distribution. Here we document the first stranding of the extant pygmy right whale in the Northern Hemisphere – on the coast of The Gambia, Africa (NE Atlantic Ocean, around latitude 13° N).

## Material and methods

**Fig. 1:**
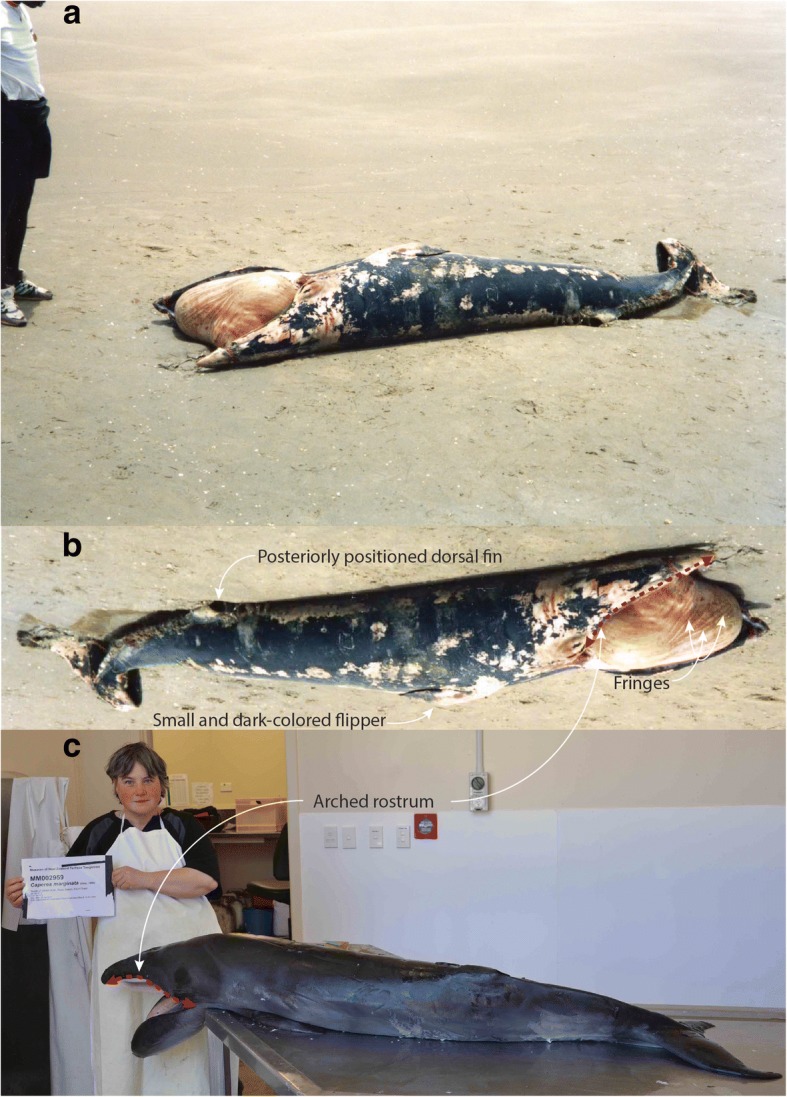
A pygmy right whale *Caperea marginata* from The Gambia, Africa. **a** The original photo of the Gambia *Caperea* by Bengt Larsen in the files of Marine Mammal Program, Department of Vertebrate Zoology, Smithsonian Institution; (**b**) The same image, edited to facilitate taxonomic comparisons (mirror image of (**a**)). **c** A stillborn *C. marginata* NMNZ MM002959 from New Zealand for comparison (photo by CHT). A key diagnostic feature, arched rostrum, is illustrated. NMNZ, National Museum of New Zealand (Te Papa Tongarewa), Wellington, New Zealand

The Gambia whale was found stranded on the beach in January 1995. Bengt Larsen photographed the specimen, as shown in Fig. 1 (the exact locality is unknown). Subsequently, Tom Arnbom (Department of Zoology, Stockholm University) mailed the photo to JGM asking for identification (see the original mail from Tom Arnbom to JGM indicating that this individual was found on the beach of The Gambia in 1995 in the Supporting Information). It seems that the stranded specimen is now missing and untraceable; the record could be found in the files of the Marine Mammal Program, Smithsonian Institution, and its identification number is STR14661 in the Cetacean Distributional Database, Department of Vertebrate Zoology, National Museum of Natural History, Smithsonian Institution, Washington DC, USA.

## Results

The photo (Fig. 1) shows sufficient diagnostic features to identify the Gambia whale as the pygmy right whale *Caperea marginata* [1, 2, 7]:

1. small body size; assuming a height of 170 cm for the person on the left (Fig. 1a), we estimate the total length of the Gambia whale to be 270 cm;
2. the overall body shape is streamlined;
3. the dorsal side of the body is generally dark-colored;
4. the rostrum is arched along its lateral margin (as illustrated in Fig. 1b);
5. triangular and narrow rostrum in dorsal view;
6. lack of head callosities;
7. some fringes on the dorsal surface of the tongue;
8. small and relatively posteriorly positioned dorsal fin;
9. small and dark-colored flipper (the skin of the proximal half had worn off).

Other diagnostic features of the pygmy right whale [1, 2, 7], including distinctive crescent-shaped pale shadings located circumferentially near the head region, a prominent median notch of the fluke, and the presence of mandibular and maxillary hairs, could not be clearly observed due to the limited visual information (only a single photo showing a dorsolateral view from the right side, as shown in Fig. 1a, b) and the preservation of the specimen (scars and rotting).

## Discussion

**Fig. 2:**
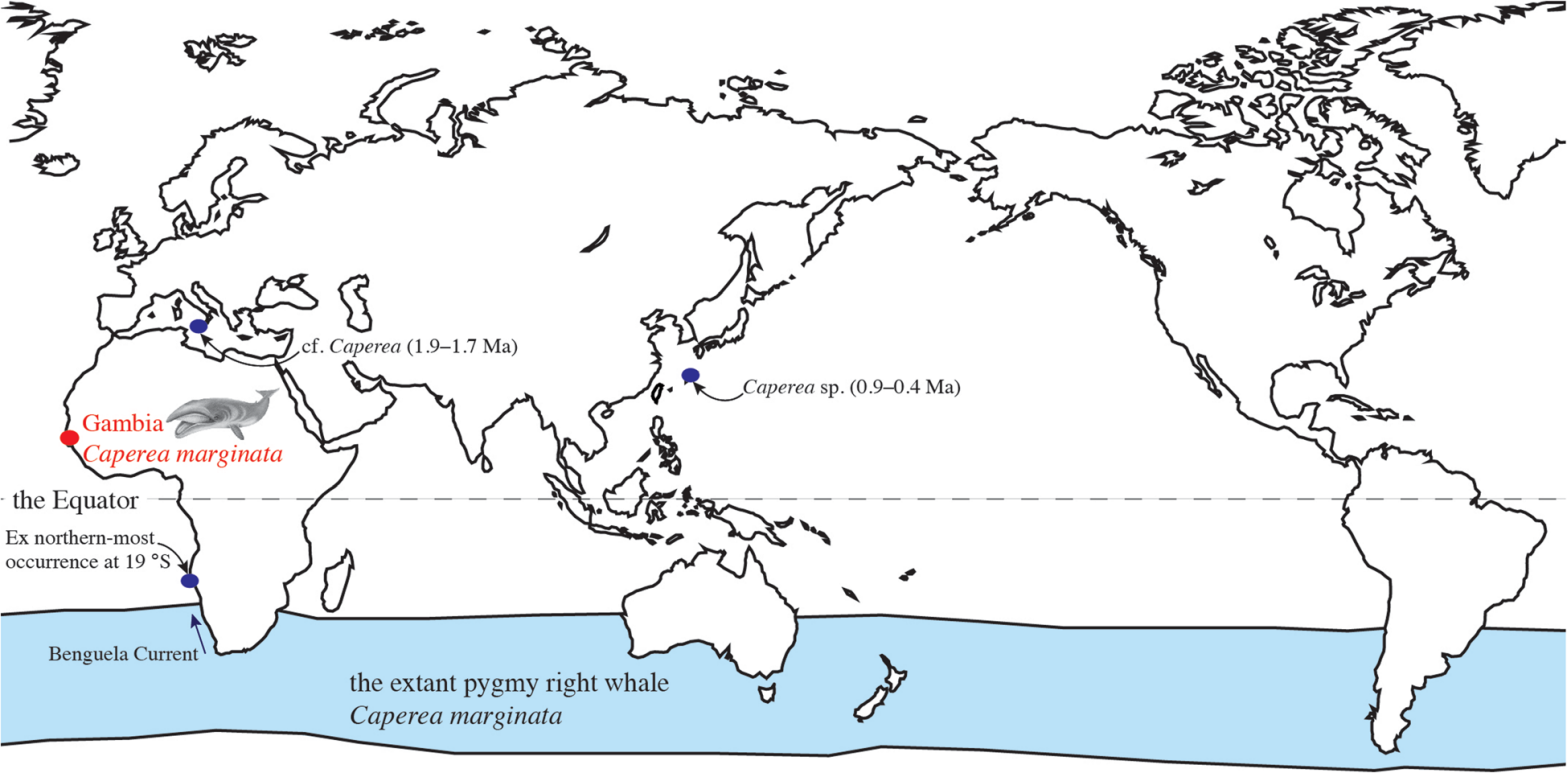
Proposed distribution of the pygmy right whale (in light blue, modified from Kemper, 2014), extralimital occurrence at 19° S, Pleistocene occurrences in the Northern Hemisphere (blue circles; Ma: million years), and the occurrence of the Gambia *Caperea* (in red; *Caperea* illustration by Moyna Müller)

Previously, the northern-most occurrence of the pygmy right whale was at 19° S (Fig. 2); an atypical sighting, likely due to drifting along the Benguela Current [2]. Our new specimen represents the first record of the extant pygmy right whale in the Northern Hemisphere (Fig. 1, NE Atlantic Ocean, around latitude 13° N, mirroring the recent description of the first *Caperea* fossils from north of the equator [6]). It remains uncertain whether this Gambia occurrence is simply a chance event, such as an already dead animal drifting northwards with the Benguela Current, ultimately washing up in The Gambia. This scenario seems more plausible than the animal actively crossing the equator, given its small size (270 cm) and, thus, rather young age. Newborn *Caperea* are around 200 cm in length [1, 2], suggesting that the Gambia whale was a young calf (less than one year old) without sufficient swimming skills for crossing the equator. Had the animal been alive during the journey, it would likely have been accompanied by its mother or a small pod; however, since 1995 (the stranding year), no other northern occurrences of *Caperea* reported.

Like the pygmy right whale, the gray whale *Eschrichtius robustus* is restricted to a single hemisphere – in this case, the North Pacific, with the North Atlantic population having gone extinct in the eighteenth century [8]. Likewise, all known fossil eschrichtiids are also from the Northern Hemisphere [9–14]. Yet, there was a single sighting of a gray whale in the Southern Hemisphere [15] – in Namibia, around latitude 13° S. This remarkable extralimital occurrence is comparable to the Gambia Caperea and, along with additional examples of equatorial crossings by other mysticete species [16, 17]), suggesting that faunal exchange between both hemispheres may be more frequent, or may be in the process of becoming more frequent, than commonly thought. Together, records such as these invite more detailed studies of how climate change and ocean currents affect the evolution and distribution of marine mammals in the context of the wider marine ecosystem [18]. In addition, they emphasize the importance of research on remote and often ignored regions for a more comprehensive picture of global changes from the past to the present and the future.

## Conclusions

Despite being a single, and likely a chance event, the first stranding of a pygmy right whale in the Northern Hemisphere is significant given the usual restriction of this species to the Southern Hemisphere. This unusual appearance of a “northern” pygmy right whale demonstrates that the biogeography of extant species is remodeling over time instead of unchangeable distribution as fossils are often found outside the recognized distribution showing how evolution is at work. Together with the current understanding of climate change and anthropogenic impact upon the globe, this unexpected find calls for more detailed studies, in particular into remote or commonly neglected areas to monitor how marine mammals are responding to global change.
